# The lack of keratinized mucosa is associated with poor peri-implant tissue health: a cross-sectional study

**DOI:** 10.1186/s40729-020-00227-5

**Published:** 2020-07-16

**Authors:** Kajorn Kungsadalpipob, Kakanang Supanimitkul, Sukuma Manopattanasoontorn, Navawan Sophon, Teerawut Tangsathian, Sirikarn P. Arunyanak

**Affiliations:** Department of Periodontology, Faculty of Dentistry, Chulalongkorn University, 34 Henry Dunant Rd., Wangmai, Patumwan, Bangkok, 10330 Thailand

**Keywords:** Keratinized mucosa, Dental implants, Peri-implantitis

## Abstract

**Objectives:**

The aim of this cross-sectional study is to determine the association between the absence of keratinized mucosa and peri-implant tissue health.

**Methods and materials:**

This cross-sectional study comprised 412 implants from 200 patients from faculty-based clinics. Demographic, medical, and clinical information were collected. The modified sulcus bleeding index, modified plaque index, mucosal recession, probing depth, bone level, the width of keratinized mucosa, and implant status were evaluated by three calibrated examiners. Each implant was categorized into either of two peri-implant mucosa groups: keratinized mucosa (KM) or non-keratinized mucosa (NKM). The chi-square test was performed to the association between the keratinized mucosa groups and peri-implant clinical parameters and peri-implant status. Multiple logistic regression models were analyzed to test potential associations between peri-implant clinical parameters and the presence or absence of keratinized mucosa.

**Results:**

Thirty-two implants (7.8%) were categorized into the NKM group. The prevalence of peri-implantitis was 12.5% and 8.3% at the subject level and implant level, respectively. The NKM group was associated with more plaque accumulation, mucosal recession, interproximal bone level ≥ 3 mm, and peri-implantitis (*p* < 0.05). After controlling for confounding factors, the NKM group demonstrated higher plaque accumulation, mucosal recession, and interproximal bone level ≥ 3 mm with adjusted odds ratios of 2.98 (1.33–6.66), 3.20 (95% CI, 1.03–9.90), and 4.62 (1.70–12.58), respectively.

**Conclusion:**

Within the limitation of this study, the lack of keratinized mucosa around the dental implants was significantly associated with more plaque accumulation, mucosal recession, interproximal bone level ≥ 3 mm, and peri-implantitis.

## Introduction

Dental implants are a successful tooth replacement treatment and are frequently requested by patients. However, like any other prosthesis, the success rate of dental implants is lower than their survival rate [1]. Dental implants may develop mechanical and biological complications, particularly peri-implantitis. Peri-implant mucositis has a prevalence of 43%, while that of peri-implantitis was 22% [2].

Dental implant success is influenced by various factors such as medical conditions, oral hygiene status, bone and soft tissue quality, and treatment factors, including the surgical procedure and prosthetic treatment. Numerous studies have reported that keratinized mucosa around dental implant functions as a barrier against microorganisms and subgingival plaque, which are potentially detrimental for the biological success of dental implants [3–7].

A classical cohort study in natural teeth by Lang and Löe recommended a minimum keratinized mucosa width of 2 mm of which 1 mm should be attached to achieve proper gingival health [8]. In contrast, several clinical studies challenged this concept, showing that gingival health could be maintained with no attached keratinized mucosa present [9–11]. However, healthy peri-implant tissues may present different histological features altering their structure, function, and resistance to bacterial infection [12–16]. Interestingly, some reports have shown no major differences in the soft tissue response to plaque deposition between dental implants and natural teeth [13, 15]. Chronic plaque accumulation induced a more rapid rate of tissue destruction in peri-implantitis lesions compared with periodontitis lesions [13, 15]. However, the role of keratinized mucosa on peri-implant tissue health has not been clarified due to disparate study outcomes.

A study evaluated the peri-implant tissue conditions at osseointegrated oral implants in relation to the keratinized mucosa width found no association between the presence of keratinized mucosa and healthy peri-implant soft tissue [17]. In contrast, a 5-year observational study reported more plaque deposition and mucosal inflammation in the absence of keratinized mucosa [5]. Furthermore, Zigdon and Machtei [7] found a negative correlation between inadequate keratinized mucosa and attachment loss and mucosal recession. Moreover, the study with larger sample size (211 subjects) reported that patients with non-keratinized mucosa demonstrated significantly more plaque accumulation and bleeding after probing compared with patients with keratinized mucosa [18]. This relationship is supported by systematic reviews [19, 20]. Lin et al. [20] conducted a systematic review of 11 studies and concluded that a lack of keratinized mucosa around dental implants was associated with increased plaque deposition, mucosal recession, mucosal inflammation, and attachment loss. In contrast, another study found no significant difference in the relation to probing depth between implants with a > 2 mm keratinized mucosa width and implants with < 2 mm keratinized mucosa width [19]. In a review literature, longitudinal studies showed no significant association between inadequate keratinized mucosa and higher plaque scores or mucosal inflammation in well-maintained populations [21]. Thus, the need of keratinized mucosa around dental implants for maintaining peri-implant health has been questioned.

Therefore, the purpose of the present study was to determine the association between the absence of keratinized mucosa and peri-implant tissue health. We analyzed peri-implant clinical parameters in a cross-sectional study design to evaluate the association between a lack of keratinized mucosa and peri-implant tissue health.

## Material and methods

### Ethics and study design

The protocol for this study was approved by the Ethical Committee, Faculty of Dentistry, Chulalongkorn University (IRB NO. 036/2017). The study followed the STROBE statements for reporting observational studies [22]. This study employed a cross-sectional design. Two-hundred patients who received endosseous dental implant at a faculty-based clinic from 1996–2014 were recruited for this study. The inclusion criteria was each patient had at least 1 dental implant restored with a fixed prosthesis that was in function for more than 1 year. The exclusion criteria was patients who had implant-supported removable prosthesis, edentulous patients, and implant-supported prosthesis was in function less than 1 year. All participating patients were placed in a maintenance program, and all gave informed consent to participate in this study.

### Data collection

The patient’s demographics, e.g., age, sex, and dental history, were obtained from history taking, past chart review, and dental examination. The data comprised their medical and dental history, smoking habits, history of periodontal therapy, oral hygiene status, history of implant treatment, and implant prosthesis types. The patients’ clinical and radiographic examinations were performed at one visit as described in a recent publication [23] and reported thoroughly in the next session. Maintenance care at the implant sites was performed according to the cumulative interceptive supportive therapy (CIST) protocol [24]. All patients received a report of their oral examination and were scheduled for their next maintenance visit.

#### Clinical examination

The clinical evaluation was performed by three calibrated examiners (NS, TT, and KS) who assessed the following clinical parameters:

- Modified plaque index (mPLI) [25]: scores were determined at 4 sites per implant (mesiobuccal, distobuccal, mid-buccal, and mid-lingual). Scored from 0 to 3: 0—no plaque detection, 1—plaque recognized by running a probe across the marginal surface of the implant, 2—plaque seen with the naked eye, and 3—abundance of soft matter.
- Modified bleeding index (mSBI) [25]: scores were determined at 4 sites per implant (mesiobuccal, distobuccal, mid-buccal, and mid-lingual). Scored from 0–3: 0—no bleeding when periodontal probe is passed along the gingival margin adjacent to the implant, 1—isolated bleeding spots visible, 2—blood forms a confluent red line on margin, and 3—heavy or profuse bleeding.
- Probing depth (PD): measurements were taken at 6 sites per implant (mesiobuccal, distobuccal, mid-buccal, mesiolingual, distolingual, and mid-lingual)
- Mucosal recession (RE): measured in millimeters from the restorative margin to the mucosal margin. Recession was measured at 6 sites per implant (mesiobuccal, distobuccal, mid-buccal, mesiolingual, distolingual, and mid-lingual).
- Width of the keratinized mucosa: the width of the keratinized mucosa was measured in millimeters at the narrowest distance between the mucosal margin and the mucogingival junction at the buccal aspect of the implant using visual and functional methodologies to identify the color, texture, and mobility differences between the keratinized mucosa and non-keratinized oral mucosa.
- Tissue phenotype: the tissue phenotype was classified as a thin phenotype if the outline of the underlying periodontal probe could be seen through the buccal mucosa, and as thick phenotype if not [26].

All measurements were manually performed with a conventional periodontal probe (UNC-15; Hu-friedy, Chicago, IL, USA) for natural teeth and a plastic periodontal probe (12-UNC COLORVUE®; Hu-Friedy, Chicago, IL, USA) for implants. Distances were measured to the nearest millimeter (mm).

#### Radiographic examination

Radiographic examination was performed using standardized periapical radiographs. Digital radiographs were then taken and visualized with Infinitt proprietary software v.2 (Infinitt Co., Seoul, Korea). The interproximal bone level (BL) was evaluated by a single calibrated examiner (TS). The interproximal bone level was defined as the distance from the implant shoulder to the alveolar bone crest and was measured at the mesial and distal aspects of each implant. The most severe bone level site was selected to represent the bone level of each implant. Because the patients had been treated with different dental implant systems, we could not define a universal point of reference for all implants. Therefore, a reference point at the abutment-crown or fixture-abutment connection was defined for the respective implant system.

Due to lacking of baseline radiographs at the insertion of prosthesis, the interproximal bone loss cannot be interpreted in this study. Therefore, the interproximal bone level will be used as a parameter for analysis.

#### Case definitions

The peri-implant health and diseases were assessed based on previously established case definitions:

- Healthy peri-implant: without peri-implant soft tissue inflammation and bone loss.
- Peri-implant mucositis: peri-implant soft tissue inflammation present with bleeding during probing at ≥ 1 aspects of the implant (recorded from the mSBI > 2) and no evidence of supporting bone loss after bone remodeling [27].
- Peri-implantitis: presence of soft tissue inflammation with bleeding and/or suppuration on probing at least 1 aspect of the dental implant (recorded from the mSBI > 2) and bone loss around an osseointegrated implant beyond functional remodeling ≥ 3 mm from time of loading [27]. When there was no baseline radiograph, a threshold vertical distance of 3 mm from the expected marginal bone level was diagnosed as peri-implantitis [28].
- Implant survival: the implant with restoration was present at the follow-up examination regardless of its condition [24].
- Keratinized mucosa: the subjects were dichotomized into two groups. The non-keratinized mucosa group (NKM) comprised patients where there was no band of keratinized mucosa present and only alveolar mucosa was detected, and the keratinized mucosa group (KM) comprised patients where the width of the keratinized mucosa was > 1 mm.
- Past periodontal status: the diagnosis of periodontal disease was classified using the American Academy of Periodontology (AAP) criteria [29]. Patients with chronic periodontitis were those with bleeding on probing and pocket depth ≥ 4 mm in at least 30% of the total sites before implant placement.
- Oral hygiene status (OHS): oral hygiene was categorized as good (mPLI < 1), fair (mPLI = 1–2), or poor (mPLI ≥ 2) [30].

### Examiner reliability

Examiner calibration was completed before the start of the study. The intra- and inter-examiner reliability of the three clinical examiners (NS, TT, and KS) were assessed using 5 volunteer non-study subjects with ≥ 1 implant restoration. Cohen’s Kappa coefficient was used as a measure of intra- and inter-examiner reliability. The mean intra- and inter-examiner Cohen’s Kappa coefficients were 0.88 and 0.86, respectively, which indicated a high degree of reliability in the measurement.

A single calibrated examiner (TS) measured the implant bone level for 30 cases randomly drawn from the database to assess the intra-examiner reliability for radiographic bone level measurement. The reassessment was performed 7 days later to determine the measurement reproducibility. The mean bone level at the first and second measurement was 1.23 ± 1.2 mm and 1.19 ± 1.18 mm, respectively, resulting in an intra-class correlation coefficient of 0.86.

### Sample size calculation

A sample size calculation was analyzed using G*Power software version 3.0.10© 1992-2008. (Universitat Kiel, Universitat Dusseldorf, Universitat Mannheim, Germany). A required sample size of 200 subjects was determined by assuming the following: (1) 90% power, (2) alpha level of 5%, and (3) a constant proportion of 0.22 was calculated from prevalence of peri-implantitis [2]. As a result of calculation, a minimum of 180 subjects was required to provide a 90 statistical power with *α* = 0.05.

### Statistical analysis

The data were analyzed using Statistical software SPSS version 22.0 (SPSS Inc, Chicago, IL, USA). The Kolmogorov-Smirnov test was performed to determine if the data for each parameter/variable was normally distributed. Descriptive statistics were reported as the prevalence of peri-implant disease at the implant level and subject level.

This study analyzed the data on the implant-based level of 412 implants. Differences in the mean clinical parameters between the NKM and KM groups were evaluated using the *t*-test or the Mann-Whitney *U* test. The chi-square test was used to evaluate the correlation between the keratinized mucosa groups (independent variables: NKM and KM) and categorical clinical parameters (mPLI, mSBI, PD, RE, BL, and implant status).

For regression model analysis, the unit of analysis was presence or absence of keratinized mucosa and peri-implant clinical parameters (presence of plaque, rececession > 1 mm, interproximal bone level > 3 mm). Univariate and multiple logistic regression analyses were performed to determine whether the absence of keratinized mucosa was associated with peri-implant clinical parameters and peri-implantitis after controlling for known confounding factors [31]: smoking, diabetes, history of periodontitis, oral hygiene status, implant location, cement or screw-retained restoration, plaque accumulation, bleeding upon probing, and probing depth > 4 mm. Statistical significance was defined as *P* < 0.05. The risk analysis is shown in terms of odds ratio (OR) with 95% confidence intervals (95% CI).

## Results

This study examined 200 patients with 412 implants whose demographic data and clinical parameters are listed in Table 1. The average patient age was 57.3 years (range 18–79 years). One hundred seventeen (58.5%) of the patients were female and 83 (41.5%) were male. The majority of the patients were nonsmokers (88%), non-diabetic (91%), and had fair oral hygiene. The prevalence of peri-implant mucositis was 27.5% at the patient level and 21.6% at the implant level, whereas that of peri-implantitis was 12.5% and 8.3%, respectively. On average, the patients had their implant in function for 4.4 years (range 1.5–15.9 years). The majority of the patients attended regular maintenance recall visits with an average 12.5-month recall interval. The long-term implant survival rate was 96% and 97.4% for the patient level and implant level, respectively.

**Table 1:** Demographic data and clinical characteristics

	Patient (Total *N* = 200) N (%)	Implant (Total *N* = 412) N (%)
Sex		
Male	83 (41.5)	151 (36.7)
Female	117 (58.5)	261 (63.3)
Smoking status		
Non-smoker	176 (88)	365 (88.6)
Former-smoker	20 (10)	42 (10.2)
Current-smoker	4 (2)	5 (1.2)
Systemic Disease		
Diabetes	18 (9)	31 (7.5)
Non-diabetes	182 (91)	381 (92.5)
Oral hygiene status		
Good	35 (17.5)	75 (18.2)
Fair	155 (77.5)	315 (76.5)
Poor	10 (5)	22 (5.3)
History of periodontal disease		
With history of periodontitis	72 (36)	174 (42.2)
Without history of periodontitis	128 (64)	238 (57.8)
Implant status		
Healthy	120 (60.0)	289 (70.1)
Peri-implant mucositis	55 (27.5)	89 (21.6)
Peri-implantitis	25 (12.5)	34 (8.3)
Location of implant		
Maxillary anterior	NA	64 (15.5)
Maxillary posterior	NA	117 (28.4)
Mandibular anterior	NA	17 (4.1)
Maxillary posterior	NA	214 (52.0)
Keratinized mucosa group		
KM	NA	380 (92.2)
NKM	NA	32 (7.8)

*NA* Non-applicable, *KM* Keratinized mucosa, *NKM* Non-keratinized mucosa

Thirty-two implants (7.8%) were placed into the NKM group, and 380 implants (92.2%) were placed into the KM group. The mean keratinized mucosa width at the implant was 2.73 ± 1.23 mm (range 1–7 mm). Overall, there was no association between presence or absence of keratinized mucosa and demographic data and clinical characteristic (supplementary table S1). Comparisons of clinical parameters (mPLI, mSBI, PD, RE, and BL) between the NKM and KM groups are presented in Table 2. There was no difference of clinical parameters between the NKM and KM groups (*p* = 0.050). Also, there was no difference between clinical parameters and these following factors; sex, location, type of prosthesis, implant system. Thin periodontal phenotype showed significant more recession than thick periodontal phenotype (*p* < 0.05) (supplementary table S2).

**Table 2:** Comparison of peri-implant clinical parameters between the NKM and KM group

	NKM group (*N* = 32)		KM group (*N* = 380)		*p*
	Min – Max	Mean ± SD	Min – Max	Mean ± SD	
mPLI	0.00 - 0.67	0.18 ± 0.25	0.00 - 2.00	0.15 ± 0.35	0.073
mSBI	0.00 - 1.33	0.25 ± 0.40	0.00 - 2.67	0.31 ± 0.46	0.446
PD	1.67 - 4.67	2.74 ± 0.64	1.67 - 8.00	2.83 ± 0.77	0.601
RE	-0.33 - 1.67	0.17 ± 0.45	-1.00 - 2.33	0.03 ± 0.26	0.050
BL	0.25 - 4.21	1.18 ± 1.43	0.20 - 7.74	0.77 ± 1.04	0.490

*KM* Keratinized mucosa, *NKM* Non-keratinized mucosa, *mPLI* modified plaque index, *mSBI* modified sulcus bleeding index, *PD* probing depth, *RE* recession, *BL* Interproximal bone level, *Min* minimum, *Max* maximum, *p* significance

**Fig. 1:**
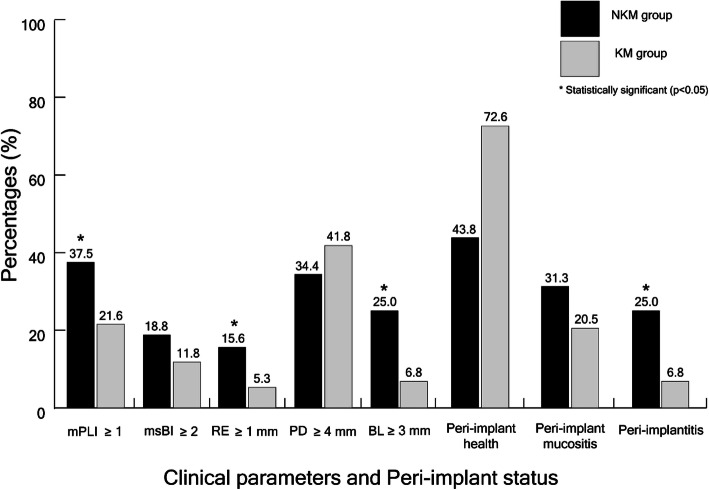
The association between the presence or absence of keratinized mucosa and clinical parameters and peri-implant status

The association between periodontal parameters and keratinized mucosa groups was determined by using the chi-square test (Fig. 1). The NKM group had a higher percentage of plaque accumulation (mPLI ≥ 1), recession (RE ≥ 1 mm), and interproximal bone level ≥ 3 mm (BL ≥ 3 mm). Similarly, the NKM group was associated with a higher percentage of peri-implantitis compared with the KM group (25% vs 6.8%). The NKM group was associated with plaque accumulation, recession, interproximal bone level ≥ 3 mm, and peri-implantitis. (*p* < 0.05). However, there was a lack of association between the absence of keratinized mucosa and bleeding or probing depth ≥ 4 mm.

The association between peri-implant clinical parameters (presence of plaque, recession > 1 mm, interproximal bone level > 3 mm) and all variables included the absence of keratinized mucosa; factors known to be associated with peri-implant disease were analyzed by multiple logistic regression analysis (Table 3). The absence of keratinized mucosa (NKM group) was significantly associated with plaque accumulation, mucosal recession, and interproximal bone level ≥ 3 mm. As a consequence of multivariate analysis, the NKM group was threefold more likely to have plaque accumulation (OR = 2.98, 95% CI, 1.33–6.66) and mucosal recession ≥ 1 mm (OR = 3.20, 95% CI 1.03–9.90).

**Table 3:** Multiple logistic regression analysis for the association between peri-implant clinical parameters and the absence of keratinized mucosa

Independent variables	N	Presence of plaque		Recession **>** 1 mm		Bone level **>** 3 mm	
		Crude OR (95% CI)	Adjusted OR (95% CI)	Crude OR (95% CI)	Adjusted OR (95% CI)	Crude OR (95% CI)	Adjusted OR (95% CI)
**Keratinized mucosa**							
**Absence of K**	**32**	2.18^*^	2.98^*^	3.33^*^	3.20^*^	4.54^*^	4.62^*^
**Presence of KM**	**380**	**(1.02-4.65)**	**(1.33-6.66)**	**(1.16-9.58)**	**(1.03-9.90)**	**(1.86-11.09)**	**(1.70-12.58)**
**Oral hygiene status**							
**Poor (mPI>2)**	**22**	1.29	1.39	0.73	0.90	1.12	0.68
**Good to fair(mPI< 2)**	**390**	(0.49-3.39)	(0.47-4.08)	(0.09-5.63)	(0.10-7.90)	(0.25-5.00)	(0.13-3.59)
**Smoking habit**							
**Smoker**	**47**	1.90	2.01	0.31	0.30	1.38	1.12
**Nonsmoker**	**365**	(0.99-3.65)	(0.99-4.03)	(0.04-2.34)	(0.04-2.35)	(0.51-3.76)	(0.38-3.33)
**Diabetes mellitus**							
**Diabetes**	**31**	1.68	1.72	1.07	1.13	1.73	1.56
**No diabetes**	**381**	(0.76-3.71)	(0.75-3.93)	(0.24-4.78)	(0.25-5.20)	(0.57-5.28)	(0.48-5.12)
**History of periodontitis**							
**Yes**	**174**	0.91	0.70	1.28	1.32	2.73*	2.45*
**No**	**238**	(0.57-1.45)	(0.42-1.17)	(0.57-2.88)	(0.57-3.10)	**(1.31-5.67)**	**(1.11-5.41)**
**Type of restoration**							
**Cement retained**	**302**	1.80^*^	1.85*	0.86	0.81	0.57	0.46
**Screw retained**	**110**	**(1.10-2.96)**	**(1.10-3.11)**	(0.33-2.21)	(0.31-2.16)	(0.23-1.40)	(0.17-1.21)
**Location of implant**							
**Posterior region**	**331**	0.96	0.76	0.76	0.67	1.46	0.99
**Anterior region**	**81**	(0.54-1.70)	(0.41-1.40)	(0.29-1.97)	(0.25-1.84)	(0.55-3.90)	(0.34-2.88)
**Bleeding on probing**							
**mSBI>2**	**51**	1.66	1.40	0.96	0.87	1.59	1.10
**mSBI < 2**	**361**	(0.87-3.15)	(0.68-2.85)	(0.28-3.34)	(0.23-3.31)	(0.62-4.04)	(0.39-3.15)
**Probing depth (PD)**							
**PD>4 mm**	**170**	2.10^*^	2.02*	1.34	1.39	3.29*	3.46^*^
**PD < 4 mm**	**242**	**(1.32-3.34)**	**(1.23-3.31)**	(0.60-3.01)	(0.59-3.24)	**(1.56-6.94)**	**(1.55-7.77)**

^*^Logistic regression analyses showed in bold face whether differences were significant (*p*<0.05)

*CI* confidence interval, *OR* odds ratio, *p* significance

The univariate logistic regression analysis revealed that the NKM group was fourfold more likely compared with the KM group to experience interproximal bone level ≥ 3 mm (Table 3). In the multivariate model, the association remained significant after adjusting for smoking, diabetes, history of periodontitis, oral hygiene status, and other potential confounding factors (OR = 4.62, CI 95% 1.70–12.58). Factors associated with interproximal bone level ≥ 3 mm were absence of keratinized mucosa, history of periodontitis, and probing depth ≥ 4 mm.

## Discussion

This cross-sectional study analyzed the association between peri-implant tissue health and a lack of keratinized mucosa. The results showed that the lack of keratinized mucosa was associated with increased plaque deposition, mucosal recession, interproximal bone level ≥ 3 mm, and peri-implantitis. These observations support the concept that non-keratinized mucosa is less resistant to insult along the implant-mucosa interface that may lead to the development

The influence of keratinized mucosa on plaque accumulation has not been clearly demonstrated in the literature. In this present study, implants without keratinized mucosa show a significant threefold higher plaque accumulation compared with implants with keratinized mucosa. This finding corresponds well with other human studies that found higher plaque scores at implants sites without keratinized mucosa [3, 4, 18, 20]. A lack of keratinized mucosa may result in an environment that is not easily cleaned and with increased susceptibility to mechanical irritation and discomfort while routine tooth cleaning procedures are performed [32].

The absence of keratinized mucosa was also associated with mucosal recession. The results from our study indicated that implants without keratinized mucosa had a significant threefold higher mucosal recession ≥ 1 mm compared with implant sites with keratinized mucosa (*p* < 0.05). This result is comparable to that of Schrott et al. [5], who found more mucosal recession on the buccal aspect of dental implants where there was a less than 2 mm width of keratinized mucosa. Zigdon and Machtei [7] showed that greater recession and less pocket formation were more often detected in regions having less keratinized mucosa. Keratinized mucosa is thought to function as a physical barrier, and its absence may make it easier for inflammation to migrate apically. However, the reasons why mucosal recession develops at the site of implant-supported restorations are controversial. Incorrect implant position, absence of keratinized mucosa, thin tissue phenotype, thin buccal bone, and reduced alveolar bone height should be considered as factors associated with mucosal recession around dental implants [33].

Due to lacking of baseline radiographs, bone loss around implants was difficult to discuss in this cross-sectional study. However, the result of analysis demonstrated that the percentage of sites with interproximal bone level ≥ 3 mm was significantly higher in the NKM group. With multiple logistic regression, implants without keratinized mucosa had significant fourfold risk of interproximal bone level ≥ 3 mm compared with sites with keratinized mucosa (*p* < 0.05). This association was significant following adjustment for smoking, diabetes, oral hygiene status, and history of periodontitis. However, this association should be carefully interpreted, because periapical radiographs can only demonstrate the interproximal bone level but the presence of keratinized mucosa was evaluated from the buccal site. Our results concur with those of several studies [34–36]. Bouri et al. [34] reported that the absence of keratinized mucosa was associated with alveolar bone loss of ≥ 2 mm and bleeding around dental implants. Conversely, Chung et al. [4] reported that there was no association between the width of keratinized mucosa and alveolar bone loss at dental implants. However, this study did not adjust for confounding variables, such as oral hygiene or smoking [4]. A systematic review also presented that there is a trend of increased bone loss at the implant sites with narrow keratinized mucosa group [20]. Therefore, more controlled studies are required to confirm the importance of keratinized mucosa on a peri-implant bone level.

Our study also evaluated the effect of keratinized mucosa on the maintenance of dental implants with regard to the presence of peri-implantitis. Interestingly, these results indicated that the NKM group was significantly associated with the presence of peri-implantitis. Our results correspond with those of Warrer et al. [6], who reported that implants placed in areas that lacked keratinized mucosa were susceptible to develop tissue breakdown. Dental implants with absent keratinized mucosa were prone to have high levels of plaque deposition with an increased incidence of peri-implantitis compared with implants with keratinized mucosa [37, 38]. In contrast, Frisch et al. [39] found no significant difference in the prevalence of peri-implantitis between implants with keratinized mucosa < 1 mm and keratinized mucosa ≥ 1 mm. A review reported a consensual association between poor oral hygiene, history of chronic periodontitis, and irregular maintenance therapy and an increased risk of peri-implantitis, while smoking and diabetes may be a potential risk factor. However, a lack of keratinized mucosa had limited evidence to be considered as a risk factor [40]. The influence of maintenance compliance on implants with a lack of keratinized mucosa has been addressed in several studies [41–43]. Increased adverse peri-implant conditions and prevalence of peri-implantitis were associated with < 2 mm keratinized mucosa in patients who were not regularly attending a minimum peri-implant maintenance therapy (PIMT) [42]. Whereas in a 5-year retrospective study where patients strictly followed maintenance schedules, a nonsignificant association was found between keratinized mucosa width and peri-implant parameters comprising marginal bone change, bleeding on probing, probing depth, and plaque index [41]. Thus, the association between the lack of keratinized mucosa and peri-implant health condition should be interpreted with caution when regular PIMT is performed.

The results of our study indicate that the absence of keratinized mucosa around dental implants was associated with more plaque accumulation, recession ≥ 1 mm, interproximal bone level ≥ 3 mm, and peri-implantitis. Several limitations of the present cross-sectional study are worth noting. First, in the current study, the initial amount of keratinized mucosa, mucosal margin, and bone level at the time of final restoration are missing. From 1996 to 2014, there was a transition period from conventional to digital radiographs at our university. Baseline radiographs, which are crucial for evaluating the level of implant bone loss at follow-up, were difficult to obtain for all patients. The result of this study can be interpreted in terms of the association not the causal relationship. Investigating alterations in peri-implant tissues over time relative to the keratinized mucosa width will be more meaningful to determine the influence of keratinized mucosa on peri-implant health. Secondly, there are many variables that could not be controlled (e.g., systemic factor, prosthesis design, operator experience) Furthermore, we performed a cross-sectional study that identified only associations between the lack of keratinized mucosa on specific peri-implant clinical parameters with various putative risk indicators. Due to the limited sample size of NKM group, a greater sample size with sufficient statistical power might be needed to identify this association in the multivariate analysis. Additional randomized controlled clinical trials are required to confirm the findings obtained in this cross-sectional study.

## Conclusions

The present findings indicated that the lack of keratinized mucosa around dental implants was associated with increased plaque accumulation, recession ≥ 1 mm, interproximal bone level ≥ 3 mm, and peri-implantitis.

## Supplementary information

**Additional file 1:** Supplementary **Table S1.** Demographic data and clinical characteristics regrading keratinized mucosa group

**Additional file 2:** supplementary **Table S2.** Comparison between peri-implant clinical parameters and other factor

## Data Availability

All data presented in manuscript are available for publication.

## Abbreviations

KM: Keratinized mucosa
NKM: Non-keratinized mucosa
mPLI: Modified plaque index
mSBI: Modified sulcus bleeding index
PD: Probing depth
RE: Recession
BL: Interproximal bone level
OR: Odds ratio
CI: Confidence intervals
