# Supplementary material for: The lack of keratinized mucosa is associated with poor peri-implant tissue health: a cross-sectional study
Source: Int J Implant Dent. 2020 Jul 16;6:28. doi: 10.1186/s40729-020-00227-5 (PMC7363759; doi:10.1186/s40729-020-00227-5)
Supplement: Supplementary file 1 — Additional file 1: Supplementary Table S1. Demographic data and clinical characteristics regrading keratinized mucosa group [file 40729_2020_227_MOESM1_ESM.docx]

|  | **NKM** | **KM** | ***p*-value** |
| --- | --- | --- | --- |
| **Sex, N (%)**  Male  Female | 9 (6)  23 (8.8) | 142 (94)  238 (91.2) | 0.297^a^ |
| **Smoking status, N (%)**  Non-smoker  Former smoker  Current smoker | 30 (8.2)  2 (4.8)  0 (0) | 335 (91.8)  40 (95.2)  5 (100) | 0.590^a^ |
| **Diabetes mellitus N (%)**  Diabetes  Non-diabetes | 2 (6.5)  30 (7.9) | 29 (93.5)  351 (92.1) | 1.000^b^ |
| **Oral hygiene status, N (%)**  Good  Fair  Poor | 4 (5.3)  28 (8.9)  0 (0) | 71 (94.7)  287 (91.1)  22 (100) | 0.220^a^ |
| **History of periodontal disease, N (%)**  Yes  No | 17 (9.8)  15 (6.3) | 157 (90.2)  223 (93.7) | 0.194^a^ |
| **Region of implant, N (%)**  Anterior region  Posterior region | 1 (1.2)  31 (9.4) | 80 (98.8)  300 (90.6) | 0.014^a^ |
| **Mucosal thickness, N (%)**  Thin periodontal phenotype  Thick periodontal phenotype | 14 (10.5)  18 (6.5) | 119 (89.5)  261 (93.5) | 0.149^a^ |
| **Type of prosthesis, N (%)**  Cement retained restoration  Screw retained restoration | 26 (8.6)  2 (2.6) | 276 (91.4)  75 (97.4) | 0.072^a^ |
| **Implant type, N (%)**  Bone level  Tissue level | 20 (7.2)  12 (8.8) | 256 (92.8)  124 (91.2) | 0.574^a^ |
| **Implant system, N (%)**  Straumann  Astra Tech  Zimmer  Replace  Others | 9 (6)  11 (8.1)  3 (5.7)  3 (15)  6 (15) | 140 (94)  125 (91.9)  50 (94.3)  17 (85)  34 (85) | 0.273^a^ |

**Table S1 Demographic data and clinical characteristics regrading keratinized mucosa group**

^a^ Differences between groups were analyzed using chi-square test

^b^ Differences between groups were analyzed using Fisher’s exact test
