# Supplementary material for: The lack of keratinized mucosa is associated with poor peri-implant tissue health: a cross-sectional study
Source: Int J Implant Dent. 2020 Jul 16;6:28. doi: 10.1186/s40729-020-00227-5 (PMC7363759; doi:10.1186/s40729-020-00227-5)
Supplement: Supplementary file 2 — Additional file 2: supplementary Table S2. Comparison between peri-implant clinical parameters and other factor [file 40729_2020_227_MOESM2_ESM.docx]

**Table S2 Comparison between peri-implant clinical parameters and other factor.**

|  | **N** | **mPLI** | **msBI** | **PD** | **RE** | **BL** |
| --- | --- | --- | --- | --- | --- | --- |
| **Sex**  **Male**  **Female** | 151  261 | 0.34 + 0.47  0.30 + 0.46 | 0.27 + 0.49  0.33 + 0.42 | 2.93 + 0.82  2.81 + 0.61 | 0.02 + 0.19  0.04 + 0.23 | 0.86 + 1.14  0.77 + 1.04 |
| **Location**  **Anterior**  **Posterior** | 81  331 | 0.28 + 0.45  0.32 + 0.46 | 0.37 + 0.45  0.29 + 0.45 | 2.72 + 0.58  2.89 + 0.71 | -0.02 + 0.18  0.04 + 0.22 | 0.95 + 0.97  0.77 + 1.10 |
| **Periodontal phenotype**  **Thin**  **Thick** | 133  279 | 0.38 + 0.48  0.29 + 0.45 | 0.34 + 0.43  0.29 + 0.46 | 2.82 + 0.57  2.88 + 0.74 | 0.77 + 0.29*  0.01 + 0.17 | 0.89 + 1.14*  0.76 + 1.05 |
| **Type of prosthesis**  **Cement retained**  **Screw retained** | 244  69 | 0.28 + 0.44  0.46 + 0.50 | 0.35 + 0.24  0.49 + 0.30 | 2.85 + 0.73  2.95 + 0.62 | 0.42 + 0.23  0.01 + 0.12 | 0.81 + 1.16  0.63 + 0.71 |
| **Implant system**  **Straumann**  **Astra Tech**  **Zimmer**  **Repla*ce***  **Other implant system** | 149  136  53  20  24 | 0.26 + 0.44  0.29 + 0.46  0.36 + 0.48  0.50 + 0.51  0.21 + 0.38 | 0.23 + 0.39  0.37 + 0.37  0.35 + 0.54  0.55 + 0.59  0.21 + 0.32 | 2.83 + 0.61  2.81 + 0.63  2.90 + 1.05  3.20 + 0.46  2.88 + 0.69 | 0.03 + 0.18  -0.01 + 0.10  -0.01 + 0.14  0.33 + 0.54**  0.11 + 0.29** | 0.65 + 1.01  0.43 + 0.66  1.18 + 1.33  1.99 + 1.64**  1.34 + 1.02** |

mPLI = modified plaque index; mSBI = modified sulcus bleeding index; PD = probing depth; RE = recession; BL = Interproximal bone level

** Significant difference (P<0.05), **Significant difference (P<0.01)*
